# Assessment of Maize Hybrids Resistance to Aspergillus Ear Rot and Aflatoxin Production in Environmental Conditions in Serbia

**DOI:** 10.3390/toxins14120887

**Published:** 2022-12-19

**Authors:** Tijana Barošević, Ferenc Bagi, Zagorka Savić, Nataša Ljubičić, Ivana Ivanović

**Affiliations:** 1 BioSense Institute, University of Novi Sad, 21000 Novi Sad, Serbia; 2 Faculty of Agriculture, University of Novi Sad, 21000 Novi Sad, Serbia

**Keywords:** *Aspergillus flavus*, aflatoxin, Aspergillus ear rot, artificial inoculation, maize hybrids, resistance, food safety

## Abstract

Aflatoxin, a naturally occurring toxin produced by the fungus *Aspergillus flavus,* is the most economically important mycotoxin in the world, with harmful effects on human and animal health. Preventive measures such as irrigation and planting dates can minimize aflatoxin contamination most years. However, no control strategy is completely effective when environmental conditions are extremely favorable for growth of the fungus. The most effective control method is growing maize hybrids with genetic resistance to aflatoxin contamination. The aim of this research was to evaluate the sensitivity of different maize hybrids to *A. flavus* infection and aflatoxin accumulation. Twenty commercial maize hybrids were evaluated in field trials with artificial inoculations using the colonized toothpicks method. The mycotoxin production potential of *A. flavus* isolates was confirmed by cluster amplification patterns (CAPs) analysis. The results of this research indicated the existence of significant differences in maize hybrids susceptibility to Aspergillus ear rot and aflatoxin B_1_ accumulation. No hybrid included in this research showed complete resistance in all conditions, but some hybrids showed partial resistance. Different hybrids also responded differently depending on the sowing date. This research showed that infection intensity is not always consistent with aflatoxin levels, and therefore visual evaluation is not enough to assess maize safety.

## 1. Introduction

Maize (*Zea mays* L.) is one of the most important agricultural species in the world, providing a staple food and is used as a source of income for many populations in developing countries [1]. Despite the clear advantages of improved varieties, the performance of a maize genotype and the potential of maize yield is largely determined by a specific combination of different factors, and is affected by abiotic and biotic stresses, such as climatic factors, pests, soil characteristics, solar radiation, field management practices, and the seed quality and genetic potential of the hybrid [2]. However, the impact of climate change in agricultural production is undoubtable. Variations in climatic conditions frequently favor the multiplication of pathogens while negatively affecting soil fertility and plant productivity. Climate change is bringing new species of diseases and pests that do not have any control methods fully developed yet [3]. The variability of climatic conditions contributes to higher biosynthesis of mycotoxins in maize, causing economic losses in production and risk for human and animal health [4]. Numerous species belonging to the genus *Aspergillus* are widely distributed worldwide, both in soil, as well as in various agricultural crops, especially maize and plant products [5]. Maize susceptibility to Aspergillus ear rot and aflatoxin accumulation presents a global economic and health problem. The most important species that causes Aspergillus ear rot is *Aspergillus flavus.* Although the species *A. flavus* is a saprophyte, under favorable conditions for development it can cause significant rotting of corn ears and kernels in the field, as well as during storage. The species *A. flavus* has the ability to synthesize aflatoxins, which are classified as the most toxic natural substances [6]. Food contaminated with aflatoxins poses a serious risk to human and animal health. Aflatoxin B_1_ is the strongest known carcinogen and is classified as a group 1 carcinogen by the International Agency for Research on Cancer [7]. Consumption of food contaminated with aflatoxin is one of the main causes of liver cancer in the world [8,9]. In addition to the carcinogenic effect, aflatoxins exhibit strong mutagenic, teratogenic, and immunosuppressive effects [10]. *A. flavus* is a xerophilic fungal species that has developed physiological mechanisms for adaptation in stressful environmental conditions [11]. High average temperatures and long dry periods lead to heat stress in plants and increased aflatoxin contamination [12,13]. Another important factor that contributes to intense appearance of *A. flavus* on corn grain are insects that represent the vector of conidia transmission, but also mechanically damage the grain, which facilitates the penetration of the pathogen into unprotected endosperm of the grain [5,14]. In order to reduce the aflatoxin occurrence in the field, preventive measures which are recommended include the selection of a suitable maize hybrid with increased tolerance to abiotic and biotic stress, timely sowing, crop rotation, proper plant nutrition, irrigation, control of insects, diseases and weeds [15,16]. The most effective measure to reduce Aspergillus ear rot and aflatoxin contamination is the cultivation of resistant maize hybrids. Therefore, numerous research studies are focused on the discovery of new sources of resistance [17,18,19,20,21,22]. There are various approaches to develop aflatoxin resistant hybrids, including molecular techniques [23]; antifungal proteins studies [24]; and studies of the morphological characteristics of ear and grain in resistant hybrids [25]. Since *A. flavus* infection is associated with drought stress, one approach is the development of drought-resistant hybrids [26,27]. Several studies have focused on the effectiveness of using insect-resistant hybrids to indirectly reduce aflatoxin accumulation [28,29]. The main difficulty in developing resistant maize hybrids to *A. flavus* is the strong interaction between genotype and environment [30]. Therefore, it is necessary to identify maize genotypes that possess stable resistance in a wide range of environmental conditions [31]. The improvement of artificial inoculation techniques could represent one of the most promising methods for the successful identification of resistant maize genotypes to Aspergillus ear rot and aflatoxin production. Therefore, the aim of this research was to evaluate the sensitivity of different maize hybrids to *A. flavus* infection and subsequent aflatoxin accumulation.

## 2. Results

### 2.1. Selection of Toxigenic A. flavus Isolates

As a result of PCR analysis, using the primer Aflafor/Bt2b, fragments of the expected size of 550 bp were obtained. The size of the fragments was the same in all tested isolates. By observing the results of electrophoresis under UV light, it was determined that all tested isolates belong to the species *A. flavus*.

**Figure 1:**
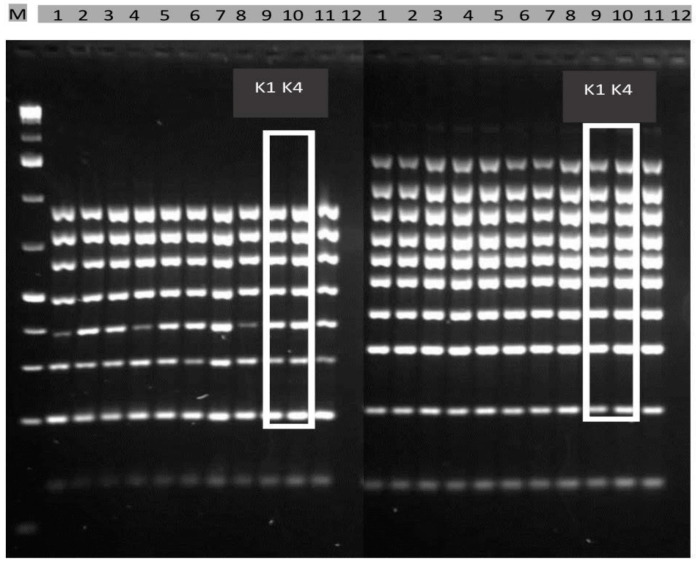
Products of multiple PCR analysis: M—1 kb Plus DNA marker; 1–8—positive control; 9—toxigenic isolate K1; 10—toxigenic isolate K4; 11—atoxigenic isolate AF36; 12—negative control [33].

From a total of 74 isolates, 20 isolates were selected for further analysis in order to test their toxigenic potential. The results of the CAPs analysis showed that two isolates were atoxigenic, while the other isolates were toxigenic (Figure 1). The amplification products were compared to a schematic diagram of chromosome 3 containing a cluster of genes responsible for aflatoxin biosynthesis [32]. No deletions on chromosome 3 were observed in the toxigenic isolates. Out of 18 toxigenic isolates, two isolates (K1 and K4) were selected for the artificial inoculation of maize hybrids, which were isolated from maize grains from the Bečej locality. Atoxigenic isolate AF36, which has all the genes responsible for aflatoxin synthesis, was used as a positive control. The atoxigenicity of this isolate is the result of a single nucleotide polymorphism (SNP).

### 2.2. Resistance of Maize Hybrids to Aspergillus Ear Rot

The results indicated different hybrid resistance to Aspergillus ear rot. Most of the ears were partially covered by fungus mycelium or without visible symptoms (ratings 1, 2, 3). A smaller number of ears had an infection rating of around 4, while ratings 5, 6 and 7 were very rare. The appearance of symptoms of Aspergillus ear rot was very rare in the non-inoculated variety, in conditions of natural infections during all three examined years.

**Figure 2:**
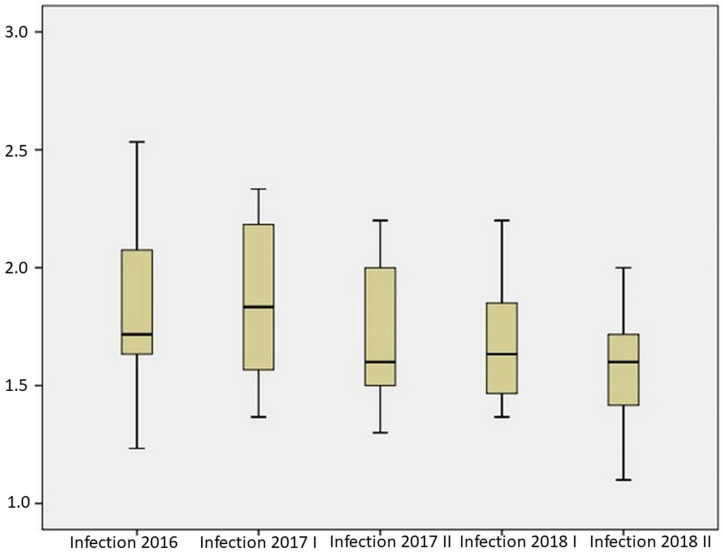
Aspergillus ear rot infection during three years of testing.

In terms of infection intensity during the three years of testing, there was an observed statistically significant difference in the infection intensity of 20 hybrids according to the testing period (chi square (4) = 21.584, *p* = 0.001). According to the obtained results, the highest average infection value was recorded in the growing season of 2017 in the first sowing period (1850), while the lowest average value was recorded in the season of 2018 in the second sowing period (1577) (Figure 2). The highest infection intensity was recorded in the season of 2016 in hybrid C50 (90% of infected ears), while the lowest infection intensity was recorded in the season of 2018 in the second sowing period in hybrid B11 (90% of healthy ears). Under artificial inoculation conditions during the first testing year (2016), the infection intensity of 20 hybrids ranged from 1.23 to 2.53. Hybrid B1 had the highest level of resistance to Aspergillus ear rot with over 70% of healthy ears. The hybrids C50, E49 and C47 had the lowest level of resistance, with over 90% of infected ears (Table 1).

The intensity of *A. flavus* infection in the second year of the study (2017) in the first sowing period varied from 1.37 to 2.33. Hybrid C25 was found to be the most resistant with over 60% of healthy ears, followed by hybrids C46 and C39, in which over 50% of ears were without visible symptoms of infection. Hybrids C12 and C16 were the most sensitive hybrids. The highest average infection intensity was recorded in this period (1850), and this was due to the fact that eight hybrids had more than 90% infected ears (C12, C16, E45, D32, E49, D26, C47, and B4). In three hybrids (E49, D32 and C16), all examined ears had symptoms of Aspergillus ear rot. Ear coverage in the second year of the study (2017), in the second sowing period, ranged from 1.30 to 2.20. In this period, hybrids C46, A15 and C25 had the highest level of resistance to Aspergillus ear rot. Hybrids C46 and A15 had over 70% healthy ears, while five hybrids had over 60% of healthy ears. Hybrids E45 and D32 had the highest susceptibility, with over 95% infected ears (Table 1).

The intensity of Aspergillus ear rot in the third year of the study (2018), in the first sowing period, varied from 1.37 to 2.20. Hybrids B11 and A15 were the most resistant, with 70% healthy ears, followed by hybrids B1 and C46, with over 60% of ears without Aspergillus ear rot symptoms. Hybrids C12 and E49 were the most sensitive, followed by hybrids C47 and A41, with over 70% infected ears. Aspergillus ear rot infection in the third year of the study (2018), in the second sowing period, fluctuated from 1.10 to 2.00. Hybrid B11 had the highest resistance with 90% of ears without symptoms of Aspergillus ear rot, followed by hybrids C46 and B10 with over 70% of healthy ears. The hybrids C47 and E49 had the highest level of sensitivity with over 70% of infected ears (Table 1).

### 2.3. Resistance of Maize Hybrids to AFB1 Production

**Figure 3:**
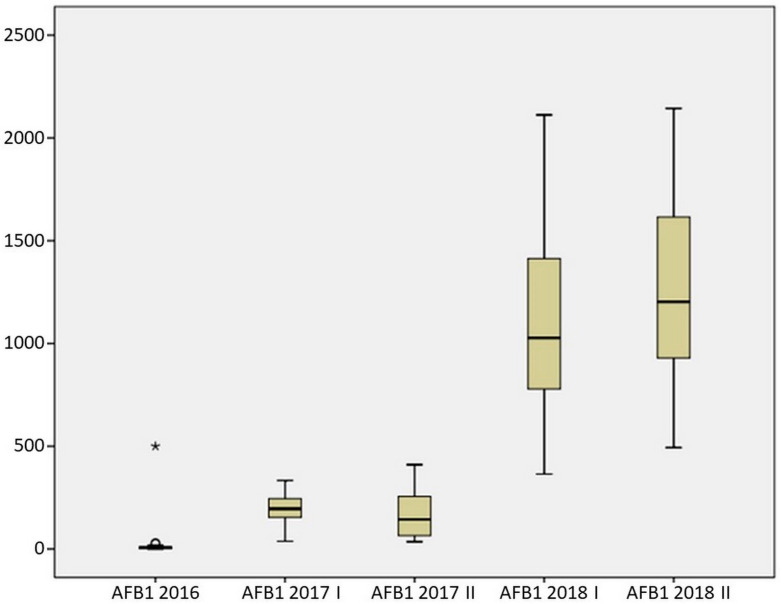
AFB1 contamination during three years of research in artificial inoculation conditions (* Hybrid A41 is outside the range of AFB1 concentrations for the other tested hybrids).

Comparing the data results regarding the amount of AFB1 in maize hybrids during three investigated years of research, there was evident statistically significant difference in the amount of AFB1 in twenty hybrids (chi square (4) = 69.920, *p* = 0.001). According to the obtained results, the highest average AFB1 concentration was recorded in the season of 2018 in the second sowing period (1272.74 µg/kg), while the lowest concentration was recorded in the season of 2016 (32.10 µg/kg) (Figure 3).

During the 2016 growing season, the average concentration of AFB1 was 32.10 µg/kg. AFB1 concentration ranged from 0 µg/kg in hybrids C25, C50, D32 to 500 µg/kg in hybrid A41. All maize hybrids were in the range of 0–28.99 µg/kg, except hybrid A41 (500 µg/kg), which affected the increase of the average AFB1 concentration for this period. AFB1 concentrations below 10 µg/kg were detected in most hybrids during this period. AFB1 was not detected in three hybrids (Table 2).

In the first sowing period of 2017, the average AFB1 concentration was 204.26 µg/kg and ranged from 37.56 µg/kg in hybrid B4 to 333.792 µg/kg in hybrid B1. The highest concentrations of AFB1 were detected in four hybrids with over 300 µg/kg of AFB1 (B1, C12, B11, A19). The lowest concentration of AFB1 was recorded in hybrid B4. In the second sowing period of 2017, the average concentration of AFB1 was 176.25 µg/kg. AFB1 concentration ranged from 35.58 µg/kg in hybrid C50 to 410.14 µg/kg in hybrid A19. The highest concentrations of AFB1 were recorded in three hybrids with over 350 µg/kg (A19, C12, C3). The lowest concentration of AFB1 was recorded in five hybrids, with aflatoxin values from 35.58 µg/kg to 55.69 µg/kg. Hybrids C50 and A41 were the most resistant. Very high concentrations of AFB1 were detected in two hybrids (C12, A19) in both sowing dates (Table 2).

In the first sowing period of 2018, the average AFB1 was 1080.55 µg/kg. The AFB1 amount ranged from 364.99 µg/kg in hybrid C3 to 2111.61 µg/kg in hybrid A41. The highest concentrations of AFB1 were detected in hybrid A41 with over 2000 µg/kg of AFB1, then in hybrids A19 and B1 with over 1500 µg/kg. The lowest concentrations of AFB1 were recorded in three hybrids (C3, B4, C47), with AFB1 values between 364.99 µg/kg and 509.48 µg/kg. In the second sowing period of 2018, the average AFB1 concentration was 1272.74 µg/kg. The AFB1 amount varied from 493.68 µg/kg in hybrid C46 to 2143.76 µg/kg in hybrid D32. Very high concentrations of AFB1 were recorded in eight hybrids (D32, A41, E45, A19, E49, B11, B4, C3), with over 1500 µg/kg of AFB1. The lowest concentrations of AFB1 were recorded in hybrids C46 and C25. In hybrids A41 and A19, very high concentrations of AFB1 were detected in both sowing dates during 2018 (Table 2).

**Figure 4:**
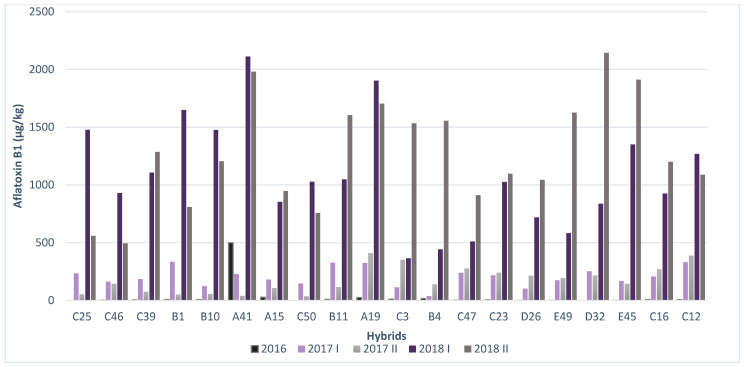
AFB1 contamination in artificial inoculation conditions across years and sowing periods.

In hybrid B4, low AFB1 concentrations were detected, particularly compared to other tested hybrids in the first sowing period in 2017 and 2018. In hybrid C25, low AFB1 concentrations were recorded compared to other hybrids in the second sowing period during 2017 and 2018, while in 2016, AFB1 was not detected in this hybrid (Figure 4).

### 2.4. Relationship between Aspergillus Ear Rot and AFB1 Levels

The results from 2016 indicate the existence of statistically significant negative correlation, of medium strength, between infection intensity and AFB1 concentration. According to data from 2017 for the first sowing period, there was not an observed statistically significant correlation between infection intensity and AFB1 levels. On the other side, the data for the second sowing period indicated the existence of a statistically significant positive correlation between infection intensity and AFB1 concentration. This data indicated that as the intensity of the infection increases, the concentration of AFB1 also increases. Based on data from the vegetation season of 2018, there was no statistically significant correlation between the observed variables for both sowing dates (Table 3). Since the existence of a statistically significant positive correlation between the intensity of Aspergillus ear rot and AFB1 concentration was determined only for the second sowing period in 2017, the visual evaluation method was not reliable enough for the quick check of a large number of hybrids. The results indicated that infection intensity is not always consistent with aflatoxin production, which indicates the existence of different mechanisms of resistance to Aspergillus ear rot and aflatoxin accumulation.

### 2.5. Influence of Sowing Date on A. flavus Infection and AFB1 Content

A higher mean infection value was recorded in the first sowing periods in 2017 (1.85) and 2018 (1.67). If we compare infection intensity across sowing dates, it can be noted that there is a statistically significant difference in the infection intensity of 20 hybrids in different sowing dates in 2017 growing season (standardized test statistic z = −3.699, *p* = 0.001). Contrary to this result, there was no statistically significant difference in the infection intensity of 20 hybrids in different sowing dates in 2018 (standardized test statistic z = −1.699, *p* = 0.089).

A higher mean AFB1 concentration in 2017 was recorded in the first sowing date (204.26 µg/kg), while the maximum AFB1 concentration was recorded in the second sowing date (410.14 µg/kg). A higher average AFB1 concentration in 2018 was recorded in the second sowing period (1272.74 µg/kg), when the highest AFB1 concentration for this period was also recorded (2143.76 µg/kg). No statistically significant difference was found in the AFB1 amount of 20 hybrids between different sowing dates in 2017 (standardized test statistic z = −0.821, *p* = 0.411) and in 2018 (standardized test statistic z = −1.232, *p* = 0.218).

The dependence between sowing date and aflatoxin B_1_ accumulation was established in both years of the study. The examined hybrids showed different sensitivity to aflatoxin B_1_ accumulation depending on the sowing period. Some hybrids were more sensitive in the first sowing period, while others showed more significant sensitivity in the second sowing period. These differences were consistent for some hybrids, while they were not uniform for others. The results confirmed that hybrid C25 had significantly higher concentrations of AFB1 in the first sowing date, while hybrid C3 had significantly higher concentrations of AFB1 in the second sowing date in both seasons.

### 2.6. Interactions between Aflatoxin B_1_ Production and Yield

The results from the 2016 growing season indicated the existence of a statistically significant correlation between aflatoxin B_1_ levels and grain yield. The correlation was strong and negative, which indicates that with an increase in the aflatoxin B_1_ amount, the yield decreases. Based on the data from 2017 and 2018, it is noted that there is no statistically significant correlation between the observed variables, which means that there are no significant differences in yields between sensitive and resistant hybrids (Table 3).

Interactions between aflatoxin B_1_ contamination and yield were confirmed, but were not consistent, since it was changeable between the years, sowing dates and depending on the hybrid. Maize hybrid B4 had the lowest concentrations of AFB1 in the first sowing period during 2017 and 2018 seasons, while in terms of yield it belonged to the group of hybrids with significantly higher yields than the others examined hybrids. In hybrid C25, the lowest concentrations of AFB1 were recorded in the 2016 season and in the second sowing period in 2017 and 2018. This hybrid was in the group of hybrids with the highest yields in 2018 and the first sowing date of 2017. Hybrid A41 had the highest concentrations of AFB1 in 2016 and 2018 and significantly higher concentrations of AFB1 compared to other hybrids in the first sowing period in 2017. It was observed that hybrid A41 established yields which were lower than average in 2016 and the second sowing period in 2017 and 2018. In hybrid A19, high concentrations of AFB1 were recorded during 2017 and 2018 in both sowing dates, and yields were lower than the average for these periods.

### 2.7. Meteorological Conditions

**Figure 5:**
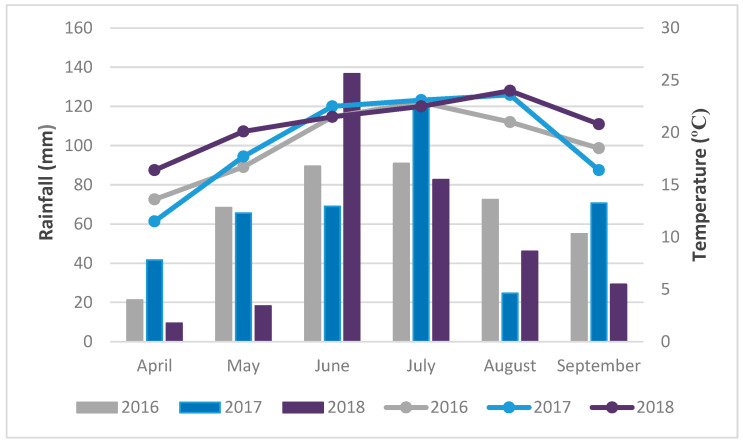
Weather characteristics of the vegetative seasons: Total monthly precipitation (in bars) and mean daily temperatures (line graph) per month during the experimental period (2016, 2017, 2018), Sombor, Serbia.

Meteorological data, precipitation and temperature, varied significantly through the growing seasons. The highest concentrations of AFB1 were recorded in 2018, when the mean air temperature was higher than the multiannual average during the entire examined period from April to September (Figure 5). High maximum air temperatures were recorded during June (33.1 °C), August (34.7 °C) and September (33.4 °C). Such high air temperatures caused heat stress in plants and favored the development of Aspergillus ear rot and aflatoxin production. A comparison of air temperature and precipitation data indicates dry conditions during August, which favored AFB1 accumulation.

High concentrations of AFB1 were also recorded during 2017, when higher mean air temperatures were recorded compared to the multiannual average from May to August (Figure 5). During June, July and August, high maximum air temperatures were recorded, ranging from 34.8 °C in June to 39.1 °C in August. Extremely dry conditions in this period favored aflatoxin production, since maize is most sensitive in the flowering and grain filling phase. The amount of precipitation was reduced compared to the multiannual average in April, June and August. The warm weather and lack of precipitation in this period contributed to reduced yields, the development of Aspergillus ear rot and aflatoxin accumulation.

Maize genotypes showed different reactions to climatic conditions, which contributes to the development of future breeding and selection of genotypes resistant to *A. flavus*, as well as unfavorable climatic conditions that favor this phenomenon.

## 3. Discussion

### 3.1. Resistance of Maize Hybrids to Aspergillus Ear Rot and Aflatoxin Accumulation

The development of resistant maize hybrids to toxigenic fungi involves different approaches, and aims to reduce ear rot and mycotoxin accumulation. Some authors examined the resistance of hybrids to ear rot, without measuring mycotoxin concentration. Brown et al. [34] examined hybrid resistance to aflatoxin accumulation, but did not consider the intensity of ear rot. Research by Campbell and White [35] combined tests of resistance to Aspergillus ear rot and aflatoxin accumulation.

Several researchers revealed a useful correlation between resistance to infection and mycotoxin contamination. Research by Henry et al. [36] showed the existence of a significant correlation between Aspergillus ear rot and aflatoxin levels. They concluded that visual testing can be a useful method for screening a large number of hybrids. Research by Walker and White [37] reported a significant correlation between infection intensity and aflatoxin levels during one year of the study, while during the second year no significant correlation between the observed variables was established. Research by Chiuraise et al. [38] confirmed the existence of a positive correlation between aflatoxin B_1_ level and Aspergillus ear rot intensity.

In this research, the existence of a statistically significant correlation between disease intensity and aflatoxin B_1_ content was determined for 2016 (negative correlation) and for the second sowing period in 2017 (positive correlation), while for 2018 and the second sowing period in 2017, no significant correlation was established. It has been already reported by several researchers [16,31,39] that resistance to Aspergillus flavus infection and aflatoxin accumulation is quantitative in nature and therefore different mechanisms probably could contribute to resistance under different environmental conditions.

The results indicated that visual evaluation is not always consistent with aflatoxin amounts. These results are confirmed by the research of Szabo et al. [40] who claim that ear rot resistance and mycotoxin resistance do not match in all cases. According to research by Mutiga et al. [41] no significant correlation was found between aflatoxin content and ear rot.

Since Aspergillus ear rot is not always consistent with aflatoxin levels, visual evaluation is not enough to assess maize safety for human and animal consumption, so measurements of aflatoxin amount are necessary.

Currently, there is no complete resistance of maize hybrids to Aspergillus ear rot and aflatoxin production, but there exists a partial resistance. Therefore, it is necessary to identify hybrids with a low risk of Aspergillus ear rot and aflatoxin accumulation. Based on disease intensity and data on aflatoxin levels, we can estimate the risk of different hybrids to contamination with toxigenic fungi [40].

No hybrid included in this research showed complete resistance in all conditions (artificial inoculation, natural infection, both sowing dates), so the risk-free category does not exist. Some hybrids showed partial resistance. In artificial inoculation conditions, low concentrations of AFB1 were detected in hybrid B4 compared to other tested hybrids in the first sowing period in 2017 and 2018. In hybrid C25, low concentrations of AFB1 were recorded compared to other hybrids in the second sowing period during 2017 and 2018, while in 2016 AFB1 was not detected in this hybrid.

In order to identify resistant and high-yielding genotypes, it is necessary to screen a large number of hybrids in different environmental conditions. Although resistance to *A. flavus* and aflatoxins is complex, selection of the best performing hybrids can accelerate the breeding process for resistance to aflatoxin production.

Interactions between maize genotype and environment are the main reason for the lack of consistency in the expression of resistance of maize genotypes to mycotoxin contamination [12,42] Therefore, it is necessary to identify maize hybrids that possess stable resistance in a wide range of environmental conditions [31,43].

### 3.2. Influence of Sowing Date on Aflatoxin B_1_ Content

Zuber and Lillehoj [44] first stated the importance of choosing the sowing date with the aim of avoiding stress during critical stages of plant development. This concept was confirmed soon after in other studies [45]. Results from studies on the influence of sowing date on the aflatoxin concentration were not consistent and found to be contrary. According to certain studies, maize from early sowing has a higher aflatoxin level, while other studies state that early sowing reduces aflatoxin levels [16].

The results from 2017 and 2018 in artificial inoculation conditions show that there is no statistically significant difference in aflatoxin concentration of 20 hybrids according to the sowing dates. Interactions between sowing date and AFB1 accumulation exist in both trial years. The tested hybrids had different sensitivity to AFB1 accumulation depending on the sowing time. Certain hybrids were found to be more sensitive in the first sowing period, while others were more sensitive in the second sowing period. These differences are consistent for some hybrids, while for others they are not. Hybrid C25 had significantly higher concentrations of AFB1 in the first sowing date, while hybrid C3 had significantly higher concentrations of AFB1 in the second sowing date in both years. In certain maize hybrids, the influence of sowing date varied depending on the year. The plant response of certain genotypes were constant in various sowing dates, whereas some other maize genotypes exposed significant variation over different sowing dates. Particularly, in the growing season of 2017 hybrid B11 had higher concentrations of AFB1 in the first sowing period, while in 2018 a higher concentration of AFB1 was recorded in the second sowing period. According to these results it becomes clear that the reason for different genotype expression of resistance to aflatoxin accumulation depending on year and sowing date is significant influence of environmental factors.

Research in North Carolina showed lower levels of aflatoxin B1 in maize planted in April compared to maize planted in May [45]. However, research by Widstrom et al. [46] show that sowing in April is at greater risk of aflatoxin contamination than sowing in May. They concluded that early crops were at greater risk because the critical period of grain filling occurs during the periods of maximum and minimum temperatures followed by the highest evaporation. These environmental conditions are most favorable to *A. flavus* infection and aflatoxin production. The influence of sowing dates across the years are in agreement with results reported by Damianidis et al. [47].

### 3.3. Natural and Artificial Resistance Tests

**Figure 6:**
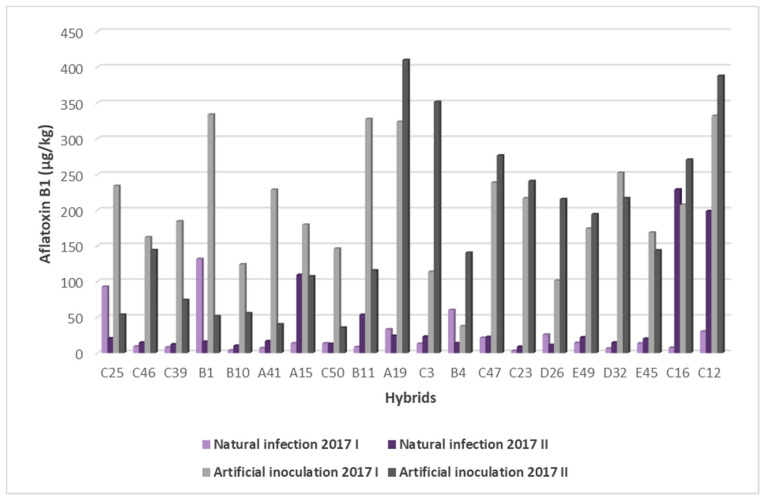
Aflatoxin accumulation (in natural and artificial inoculation conditions) in maize 2017.

The method of artificial inoculation by inserting a toothpick into a corn ear proved to be effective, because it caused a significantly higher AFB1 concentration compared to the non-inoculated variant. Average concentrations of aflatoxin B_1_ were five to seven times higher during 2018 under natural infection conditions as well as under artificial inoculation conditions compared to 2017. With this method, the symptoms of infection are more pronounced and the differentiation of hybrids according to resistance is better compared to methods without ear damage. When a toothpick is inserted into the ear, the grain is damaged, ensuring fungal entry. According to Löffler et al. [48] the colonized toothpick method gives more stable results than the inoculation method through the silk channel. During 2017, hybrids C16 and C12 had high levels of aflatoxins both in natural and artificial resistance tests. Other hybrids showed different resistance in natural and artificial conditions. These differences are related to the different resistance mechanisms of the hybrids (Figure 6). Natural resistance tests allow the identification of maize genotypes whose resistance mechanism is located on the grain surface such as wax and pericarp. Using wounding inoculation techniques, it is possible to identify genotypes with internal resistance mechanisms [22].

Natural infections of ears with *A. flavus* species occur sporadically from year to year and often can provide wrong data in the assessment of hybrid resistance to aflatoxins [49]. Artificial inoculation methods have been developed in order to uniformly infect ears with *A. flavus* [50]. The development of artificial inoculation techniques is complicated, because *A. flavus* is a weak pathogen. Difficulties are also created by environmental conditions, which have a significant impact on grain infection and aflatoxin accumulation.

In the present study, a mixture of two toxigenic isolates of *A. flavus* was used. Isolates can differ in terms of aggressiveness and aflatoxin production. Due to the pronounced divergence in terms of aflatoxin biosynthesis within the *A. flavus* population, the toxigenic potential of isolates was confirmed using cluster amplification pattern (CAP) analysis according to the method of Callicot and Cotty [32].

Development of artificial inoculation techniques that separate resistant from susceptible hybrids regardless of environmental conditions is a priority. Wounding techniques are generally less sensitive to environmental factors than non-wounding techniques. The application of non-wounding inoculation technique can result in a lower degree of infection with *A. flavus* and a lower concentration of aflatoxin when environmental conditions are not favorable for fungus development [51].

### 3.4. Relationship between Yield and Aflatoxin B_1_ Concentration

A statistically significant correlation between aflatoxin B_1_ concentration and yield was established in 2016. The correlation was strong and negative, which means that as the amount of aflatoxin B_1_ increases, the grain yield decreases. However, during 2017 and 2018, a statistically significant correlation between the observed variables was not established. This result was expected since maize genotypes have varied reactions in different years, treatments or the combination of these factors [52]. However, grain weight, as an important grain yield component, has been controlled by a number of minor genes and as a quantitative trait under the influence of environmental conditions. Interactions between aflatoxin contamination and yield were observed, but were not consistent, and were found to be changeable between years, sowing dates, depending on the hybrid. Maize hybrid B4 showed the lowest concentrations of AFB1 in the first sowing period during 2017 and 2018 growing seasons, while in terms of yield it belonged to the group of hybrids with significantly higher yields than the others examined. In hybrid C25, the lowest concentrations of AFB1 were recorded in 2016, as well as in the second sowing period of 2017 and 2018 season. This hybrid belongs to the group of hybrids with the highest yields in 2018 and the first sowing date of 2017. These results are expected since grain yield potential is the final product of plant growth and development and several complex factors, such as biotic and abiotic stress tolerance, adaptation to different soils, climate changes, and disease resistance significantly contribute to plant productivity [53].

The results of a ten-year study by Wahl et al. [21] also showed that there is no consistent relationship between yield and aflatoxin amount. In 2006, a significant negative correlation was recorded between the yield and the aflatoxin amount, while in 2009 and 2014, a significant positive correlation was recorded. Certain maize hybrids have shown extremely low aflatoxin content during many years of testing, but also low yields. Several hybrids had low aflatoxin concentrations and relatively high yields. However, these results indicated that mycotoxin production is more dependent on weather conditions than the distribution of toxigenic fungal species. Research by Betran et al. [54] reported a significant negative correlation between yield and aflatoxin amount. Significant negative correlation between aflatoxin concentration and maize yield only during an extremely dry year was observed by Damianidis et al. [47].

## 4. Conclusions

In light of the present findings it can be concluded that there was a significant difference in the relationship between the aflatoxin content and grain yield, which was variable, depending on maize genotype and environmental conditions. In this research, some hybrids showed partial resistance to aflatoxin accumulation. Maize hybrids that had low concentrations of aflatoxin may have internal or external kernel characteristics that have not been identified in hybrids previously reported to be resistant to *A. flavus.* The levels of resistance in these hybrids varied depending on the sowing period, but also on whether the hybrid was tested under artificial inoculation conditions or natural infection. In order to reduce the risk of aflatoxin contamination, it is necessary to adapt the sowing period to a particular hybrid. Most hybrids showed different levels of resistance in natural and artificial conditions. Therefore, it is necessary to constantly improve artificial inoculation techniques to identify hybrids with different resistance mechanisms. The results of this research showed that visual evaluation is not always consistent with aflatoxin accumulation. Therefore, visual rating of the hybrids is not enough to estimate food or feed safety, and direct aflatoxin measurements are required.

## 5. Materials and Methods

### 5.1. Selection of A. flavus Isolates

Two toxigenic isolates of *A. flavus* were used for artificial inoculation of maize hybrids. In order to select toxigenic isolates of *A. flavus*, 74 isolates previously identified on the basis of morphological characteristics were subjected to PCR analysis to confirm identification. The isolates were grown on malt extract agar (MEA) medium and incubated at 25 °C for 7 days. For DNA extraction the isolates were subcultured in YPD broth and incubated at 25 °C for 2 days. The species-specific primer Aflafor and universal reverse Bt2b primer were used for PCR analyses [55]. The reaction mixture contained: 2 µL of DreamTaq Buffer, 4 µL of dNTP mix, 2 µL of each primer, 5 µL of DNA-free water, 0.2 µL of DreamTaq DNA polymerase and 1 µL DNA template. PCR reactions were performed with the following cycling protocol: 3 min at 94 °C; 35 cycles of 30 s 94 °C, 30 s 64 °C, 20 s 72 °C; followed by 2 min at 72 °C. PCR products were observed on 1% agarose gel in 0.5 × TAE buffer.

### 5.2. Examination of Toxigenic Potential of A. flavus Isolates

Due to the pronounced divergence in aflatoxin biosynthesis within *A. flavus* population, the toxigenic potential of the isolates was examined by Cluster Amplification Patterns (CAPs) analysis according to the method by Callicott and Cotty [32]. CAPs analysis included four multiple PCR reactions that amplify 32 markers. Each reaction consisted of: 0.08 µmol^−1^ of each primer, 1 × AccuStart II PCR SuperMix (Quanta Biosciences, Gaithersburg, MD, USA) and 6 ng genomic DNA. The samples were subjected to the following PCR programs: 94 °C for 1 min, followed by 30 cycles of 94 °C for 30 s, 62 °C for 90 s, 72 °C for 90 s and final extension at 72 °C for 10 min. PCR products were observed on 1.4% agarose in 1 × sodium boric acid buffer [35].

### 5.3. Inoculum Preparation

Toothpicks were boiled in water three times to remove tannins and other substances that inhibit fungal growth. After that, the toothpicks were air-dried. PD broth was used as a substrate (200 g of potatoes, 20 g of dextrose in 1 L of water). PD broth with toothpicks was autoclaved. Two fragments of two toxigenic isolates of *A. flavus* were put in the substrate and incubated at 31 °C. The isolates were obtained from maize seeds originating from locality Bečej (Serbia).

### 5.4. Experimental Design and Artificial Inoculation of Maize Ears

The present investigation was carried out at the experimental trial field in Sombor (Vojvodina region, Serbia), during three growing seasons of 2016, 2017 and 2018. The experimental material in this study was comprised of twenty commercial maize hybrids (*Zea mays* L.) of different maturity classes and vegetation period which were selected and evaluated for resistance to ear rot and aflatoxin accumulation in field trials with artificial inoculations using colonized toothpicks method. In the first vegetation season of 2016 the sowing was completed on 18 April. During the seasons of 2017 and 2018, twenty maize hybrids were planted in two sowing dates, early and late. Sowing was done on 18 April, 12 May in 2017, and 26 April and 5 May in the season of 2018. The experimental trials were conducted according to the randomized complete block design (RCBD) with four replications. Conventional cultural practices were applied in all the test plots. Monitoring of maize during the flowering was performed in order to determine the date of artificial inoculation. Inoculation was performed 10–14 days after 50% of plants entered the flowering stage. An infected toothpick was inserted through the hole made by awl. In the inoculated variants of each hybrid, all ears on each plant were infected.

### 5.5. Visual Evaluation of Disease

Visual evaluation of disease intensity was performed according to the method of Reid et al. [56]. Infection intensity was visually rated using a scale of 1 to 7. Each ear is ranked on the scale according to the percentage of infected kernels labeled as follows: 1 represents completed absence of symptoms, 2 represents 1–3% infected kernels, 3 represents 4–10% infected kernels, 4 represents 11–25% infected kernels, 5 represents 26–50% infected kernels, 6 represents 51–75% of infected kernels, 7 represents 76–100% infected kernels. During each growing season of research, 80 inoculated ears per hybrid were examined (20 ears in four replicates). In the uninoculated variant, 60 ears per hybrid were tested (15 ears in four replicates), while the examined ears were selected randomly.

### 5.6. Harvest and Sampling

In each vegetation season, inoculated ears were harvested at full maturity when the kernel reached 14% or less grain moisture, expressed as total grain weight per plot after harvesting. During harvest, maize grain yield was measured while samples of each hybrid were collected for aflatoxin B_1_ analysis.

### 5.7. Aflatoxin B_1_ Measurement

The ground sample (20 g) was extracted with 100 mL of 70% methanol solution and homogenized in an Ultra Turrax T18 homogenizer (IKA, Staufen, Germany) at 11,000 rpm for 3 min. After extraction and filtration through filter paper (Filtros Anoia, Barcelona, Spain), the sample is ready for analysis. AgraQuant^®^Aflatoxin B_1_ Test Kit (Romer Labs, Tulln, Austria) was used to determine the concentration of aflatoxin B_1_. The procedure was carried out according to the manufacturer’s instructions: 200 µL conjugate was added into each color-coded dilution well; 100 µL samples was added to the conjugate; 100 µL content was transfered to antibody-coated wells; the content was incubated for 5–60 min; contents were discarded from the wells and wells were washed with deionized water; 100 µL substrate was added into each well; incubation were performed for 5–20 min; 100 µL stop solution was added into each well. Quantification of the concentration of aflatoxin B_1_ was performed using an ELISA reader at a wavelength of 450 nm (BioTec Instruments, Winooski, VT, USA).

### 5.8. Meteorological Conditions

Meteorological factors represent one of the most significant external factors that have a great influence on the development of Aspergillus ear rot and aflatoxin synthesis [4,29]. The meteorological conditions of vegetation seasons during the trial were obtained from automatic weather stations of the Republic Hydrometeorological service of Serbia. Monthly precipitation and average temperature were collected from the weather station (Metos^®^, Pessl Instruments, Weiz, Austria) located near the experimental fields, in Sombor, Serbia.

### 5.9. Data Analysis

All statistical tests used in the analysis belong to the group of non-parametric tests that do not assume the shape of data distribution. Spearman’s rank correlation tests were used for correlation analyses between Aspergillus ear rot, aflatoxin B_1_ accumulation, and grain yield. Friedman test was performed to determine differences in aflatoxin production of 20 hybrids according to the test period. Wilcoxon signed rank test was used to determine differences between hybrids according to the sowing dates. All statistical analyses were carried out using STATISTICA software, version 13 (StatSoft Inc., Tulsa, OK, USA).

## Figures and Tables

**Table 1:** Intensity of Aspergillus ear rot across years and sowing dates.

Maize Hybrids	2016	2017 I	2017 II	2018 I	2018 II
C 25	1.733333	1.366667	1.33333333	1.866667	1.500000
C 46	2.233333	1.433333	1.3	1.433333	1.233333
C 39	2.033333	1.433333	1.5	1.566667	1.366667
B 1	1.233333	1.466667	1.43333333	1.433333	1.366667
B 10	1.600000	1.566667	1.5	1.833333	1.300000
A 41	2.050000	1.566667	1.5	1.933333	1.566667
A 15	1.650000	1.600000	1.33333333	1.366667	1.633333
C 50	2.533333	1.633333	1.53333333	1.733333	1.466667
B 11	1.633333	1.633333	1.5	1.366667	1.100000
A 19	1.675000	1.700000	1.5	1.633333	1.500000
C 3	1.500000	1.966667	1.96666667	1.766667	1.633333
B 4	1.500000	1.966667	1.66666667	1.466667	1.633333
C 47	2.233333	2.033333	2	1.933333	2.000000
C 23	1.700000	2.033333	1.93333333	1.633333	1.866667
D 26	1.733333	2.100000	2	1.466667	1.566667
E 49	2.300000	2.266667	2	2.000000	1.966667
D 32	1.900000	2.266667	2.16666667	1.600000	1.733333
E 45	2.100000	2.300000	2.2	1.600000	1.700000
C 16	1.666667	2.333333	1.93333333	1.700000	1.633333
C 12	1.633333	2.333333	2.1	2.200000	1.766667
Mean	1.832	1.850	1.720	1.677	1.577
Median	1.717	1.833	1.600	1.633	1.600
Standard deviation	0.327	0.343	0.308	0.230	0.234
Rank	1.300	0.967	0.900	0.833	0.900
Minimum	1.233	1.367	1.300	1.367	1.100
Maximum	2.533	2.333	2.200	2.200	2.000

Highlighted data: values lower than mean for the given period.

**Table 2:** AFB1 accumulation (µg/kg) following artificial inoculation of *A. flavus* in maize hybrids across years and sowing dates.

	Growing Seasons				
Maize Hybrid	2016	2017 I	2017 II	2018 I	2018 II
C 25	0	233.95	53.6175	1476.9125	559.3425
C 46	2.48	161.9825	143.9425	930.215	493.68
C 39	4.85	184.375	74.205	1107.78	1286.35
B 1	8.08	333.7925	51.41	1648.055	809.14
B 10	7.82	124.08	55.6975	1476.325	1205.1325
A 41	500	228.4925	40.0525	2111.61	1979.7575
A 15	28.99	179.51	107.3	853.2225	947.785
C 50	0	146.0575	35.582	1027.83	757.095
B 11	9.1	327.4225	115.635	1047.49	1604.42
A 19	25.6	323.5975	410.1375	1902.5925	1703.31
C 3	11.71	113.795	351.475	364.99	1533.66
B 4	16.29	37.56	140.285	442.5625	1554.66
C 47	2.28	238.665	276.39	509.4825	910.3525
C 23	4.76	216.655	240.7575	1026.67	1096.56
D 26	1.22	101.245	215.42	719.6175	1043.0775
E 49	1.69	174.1225	194.2375	582.4	1626.24
D 32	0	252.1225	216.74	838.0125	2143.7625
E 45	1.31	168.5925	143.6325	1350.485	1910.97
C 16	8.66	207.3725	270.575	925.6625	1200.4325
C 12	7.17	331.885	387.9375	1269.0225	1089.01
Mean	32.101	204.264	176.252	1080.547	1272.737
Median	6.010	195.874	143.787	1027.250	1202.782
Standard deviation	110.427	82.386	117.549	474.133	469.879
Rank	500.000	296.232	374.556	1746.620	1650.082
Minimum	0.000	37.560	35.582	364.990	493.680
Maximum	500.000	333.792	410.138	2111.610	2143.762

Highlighted data: values lower than average for the given period.

**Table 3:** Correlations between infection intensity, yield and AFB1 accumulation across years and sowing dates.

Examination Period	Correlations	Infection Intensity	Yield	AFB1
2016	Infection intensity	1.000	0.787 ** 0.000	−0.588 ** 0.006
	Yield	0.787 ** 0.000	1.000	−0.736 ** 0.000
	AFB1	−0.588 ** 0.006	−0.736 ** 0.000	1.000
2017 I	Infection intensity	1.000	0.442 0.051	−0.002 0.992
	Yield	0.442 0.051	1.000	0.074 0.755
	AFB1	−0.002 0.992	0.074 0.755	1.000
2017 II	Infection intensity	1.000	0.692 ** 0.001	0.548 * 0.012
	Yield	0.692 ** 0.001	1.000	0.225 0.340
	AFB1	0.548 * 0.012	0.225 0.340	1.000
2018 I	Infection intensity	1.000	0.302 0.196	0.081 0.733
	Yield	0.302 0.196	1.000	0.329 0.157
	AFB1	0.081 0.733	0.329 0.157	1.000
2018 II	Infection intensity	1.000	0.086 0.718	0.233 0.323
	Yield	0.086 0.718	1.000	0.140 0.556
	AFB1	0.233 0.323	0.140 0.556	1.000

** *p* = 0.01, * *p* = 0.05.

## Data Availability

The data presented in this study are available in this article.
